# Effects of mavoglurant on visual attention and pupil reactivity while viewing photographs of faces in Fragile X Syndrome

**DOI:** 10.1371/journal.pone.0209984

**Published:** 2019-01-17

**Authors:** David Hessl, Danielle Harvey, Stephanie Sansone, Crystal Crestodina, Jamie Chin, Reshma Joshi, Randi J. Hagerman, Elizabeth Berry‐Kravis

**Affiliations:** 1 MIND Institute, University of California Davis Medical Center, Sacramento, California, United States of America; 2 Department of Psychiatry and Behavioral Sciences, University of California Davis School of Medicine, Sacramento, California, United States of America; 3 Division of Biostatistics, Department of Public Health Sciences, University of California Davis School of Medicine, Davis, CA, United States of America; 4 Department of Pediatrics, Rush University Medical Center, Chicago, IL, United States of America; 5 Department of Pediatrics, University of California Davis School of Medicine, Sacramento, California, United States of America; 6 Department of Neurological Sciences, Rush University Medical Center, Chicago, IL, United States of America; 7 Department of Biochemistry, Rush University Medical Center, Chicago, IL, United States of America; TNO, NETHERLANDS

## Abstract

**Background:**

Numerous preclinical studies have supported the theory that enhanced activation of mGluR5 signaling, due to the absence or reduction of the *FMR1* protein, contributes to cognitive and behavioral deficits in patients with fragile X syndrome (FXS). However multiple phase 2 controlled trials in patients with FXS have failed to demonstrate efficacy of compounds that negatively modulate mGluR5, including two phase 2b randomized controlled trials (RCT) of mavoglurant (AFQ056, Novartis Pharma AG), when the primary measures of interest were behavioral ratings. This has cast some doubt onto the translation of the mGluR5 theory from animal models to humans with the disorder.

**Methods:**

We evaluated social gaze behavior–a key phenotypic feature of the disorder—and sympathetic nervous system influence on pupil size using a previously-validated eye tracking paradigm as a biobehavioral probe, in 57 adolescent or adult patients with FXS at baseline and following three months of blinded treatment with one of three doses of mavoglurant or placebo, within the context of the AFQ056 RCTs.

**Results:**

Patients with FXS treated with mavoglurant demonstrated increased total absolute looking time and number of fixations to the eye region while viewing human faces relative to baseline, and compared to those treated with placebo. In addition, patients had greater pupil reactivity to faces relative to baseline following mavoglurant treatment compared to placebo.

**Discussion:**

The study shows that negative modulation of mGluR5 activity improves eye gaze behavior and alters sympathetically-driven reactivity to faces in patients with FXS, providing preliminary evidence of this drug’s impact on behavior in humans with the disorder.

## Introduction

Over the past decade, fragile X syndrome (FXS) has been at the forefront of translational efforts in neurodevelopmental disorders to bring targeted treatments from basic laboratory studies and animal models to patients and their families, with the goal of normalizing the neurobiology, behavioral disturbances and cognitive deficits associated with the Fragile X Mental Retardation 1 (*FMR1*) gene mutation. Despite unprecedented efforts by numerous laboratories and multinational clinical teams, several large phase 2b controlled trials have failed to demonstrate clinical benefits of these treatments [1–4], and the field is currently re-evaluating these models and trial designs to prepare for a “second wave” of novel treatments for FXS and related conditions [5–7]. A thorough and deep analysis of the data arising from the recent trials is crucial in determining whether target engagement was achieved, and for guiding the study design retooling efforts. Here, we report evidence that mavoglurant (AFQ056, Novartis), an mGluR5 negative allostatic modulator, improves a core phenotypic feature of FXS in the laboratory, despite its failure to show significant behavioral improvement over placebo, in two Phase 2b trials of this compound [2].

The Fragile X Mental Retardation Protein (FMRP) is an mRNA binding protein that aids in regulating the translation of many neuronal mRNA [8]. The absence of this protein leads to dysregulation of translation of these mRNAs and abnormal levels of their protein products, contributing to substantial deficits in synaptic function and brain development. FXS is an X chromosome-linked genetic condition associated with significant reduction or complete absence of FMRP. The phenotypic expression among those with the full mutation is quite varied and consists of physical features, intellectual disability, comorbid autism or autistic like behaviors, as well as high rates of anxiety and social withdrawal [9–14], inattention and distractibility [15–19], disinhibition and impulsivity [16, 20], hyperactivity [11, 21–23], aggression [24], and self-injury [24–26].

Research with animals models of FXS (e.g. *Fmr1* knockout mice) has demonstrated cellular abnormalities in class I metabotropic glutamate signaling (mGluR; [27, 28]) resulting in some of the phenotypic features associated with FXS. This discovery has been commonly referred to as the “mGluR theory of FXS” although it is widely recognized that there is abnormal signaling of a broad array of receptors and pathways in the absence of FMRP, in addition to group 1 mGluRs. Following the proposal and growing acceptance of this theory, there was a surge of studies examining several FXS-targeted pharmacological treatments. Results from ensuing pre-clinical work with animal models of FXS published in over 40 papers in the literature found reversal of numerous phenotypic features following pharmacological treatment with mGluR5 negative modulators in both the fly and mouse models [28–32] and following genetic reduction of mGluR5 activity in *fmr1* knockout mice also heterozygous for knockout of the mGluR5 gene. Yet, despite such compelling early evidence, translation to clinical trials with humans has seen limited success [2, 4, 33]. Many factors may contribute to these contradictory findings, possibly including differences in genetic and environmental variability between animal models and humans with FXS, potentially differing developmental windows at which targeted treatment might be effective, and lack of focus on measurement of the core problem of plasticity with cognition and learning outcomes [34].

More recently, discussion has focused on the lack of adequate research on outcome measures that have proven feasibility, reliability, and sensitivity to the core phenotypic features of FXS [35] as a potential factor in the failure of targeted treatment trials. Indeed, the trials relied on symptom-specific caregiver rating scales originally developed in more heterogeneous clinical populations such as intellectual disability and autism spectrum disorder [6]. Molecularly targeted treatments differ from traditional symptom-based approaches; as such the outcome measures used in targeted treatment trials may need to be more syndrome-specific and closer to the underlying neurobiology of the condition, at least at the stage of determining whether there is target engagement.

Gaze avoidance is a hallmark phenotypic feature of FXS that reflects social anxiety and hyperarousal, and interferes with and alters social reciprocity, engagement and social-emotional development [36–41]. The functional neuroanatomy of aberrant gaze in FXS is fairly well-described, involving alterations in frontal and medial temporal regions underlying social cognition and emotion [39, 40, 42–44]. Using an infrared eye-tracker, we previously developed a paradigm to objectively measure this aspect of the phenotype and have demonstrated that individuals with FXS make fewer gaze fixations and reduced looking time to the eye region of human faces, and greater pupil reactivity to emotional faces, when compared to typically developing controls [41]. Furthermore, test-retest data from this paradigm showed that these measures were highly reliable across a period of approximately two weeks [38], suggesting that they may be useful biobehavioral outcome measures for early stage clinical trials to demonstrate target engagement.

To examine this possibility, we examined eye gaze behavior using the paradigm at baseline and following 12 weeks of randomized, double-blind treatment with mavoglurant (Novartis AG, Basel, Switzerland), an mGluR5 negative modulator investigated as a possible targeted treatment for FXS, or identical placebo. We hypothesized that treatment with mavoglurant would be associated with increased looking time and fixations to the eye region relative to baseline, and relative to the placebo control group, regardless of the emotional valence of the stimuli, as well as reduced pupil dilation relative to baseline when viewing emotional faces.

## Materials and methods

### Participants

Participants in this study were diagnosed with FXS with molecular confirmation, and enrolled in one of two identically designed randomized, double-blind, RCTs of mavoglurant, with the only difference between the trials being that one trial enrolled adolescents age 12–17, and the other enrolled adults age 18–45. The clinical trials, taking place at 31 institutions in 16 countries, were registered in clinicaltrials.gov with the identifiers NCT01253629 and NCT01357239. The adult study was initiated in November 2010 and completed in August 2013, whereas the adolescent study was initiated in May 2011 and completed in January 2014. The full results of these trials were previously reported by Berry-Kravis and colleagues [2]. We obtained permission from Novartis to employ the eye tracking protocol as an outcome measure at our two institutions, University of California Davis MIND Institute (UCD) or Rush University Medical Center (RUMC) and we established the hypotheses prior to initiation of the two trials (rather than a *post-hoc* analysis). The UCD and RUMC IRBs approved this study. Participants were between 12 and 45 years old, had an IQ below 70, and as part of the trial were randomly assigned to receive either one of three doses of mavoglurant (25mg, 50mg, or 100mg) or placebo. These doses were chosen by Novartis based on receptor occupancy at these doses in a PET study in normal controls which ranged from about 27% (25 mg) to around 59% (100 mg)–it was thought that this amount of negative modulation of mGluR5 receptor would cover the range that might be effective in FXS based on preclinical studies. In these animal studies, with a mouse crossed with the mGlur5 het, it was assumed that 50% inhibition was achieved; thus Novartis was targeting 50% inhibition in the trial. However, this of course is based on receptor occupancy targets and not clinical response, and the optimal receptor occupancy in patients with FXS is not known. A full description of the clinical-trial design and results based on the pre-determined primary endpoints is described in Berry-Kravis et al. [2]. Sixty-six of the original 314 total participants randomized into the mavoglurant trials completed the eye tracking paradigm described below during two visits: prior to randomization at the baseline visit and at the end of the placebo-controlled period after receiving three months of treatment with study drug. All participants or their guardians provided written consent and assent was obtained from participants when possible. The following results are based on an analysis of this subsample of individuals who were enrolled at either RUSH (n = 35) or UCD (n = 31) sites where eye tracking data were collected. Among the 66 participants, n = 17 were randomized to 100 mg, n = 16 to 50 mg, n = 13 to 25 mg, and n = 20 to placebo arms in the trial. However, 9 participants were unable to provide eye tracking data due to behavioral difficulties or scheduling limitations, yielding a final sample of 57 for final analyses (n = 18 placebo; n = 39 mavoglurant). The age range, IQ level and total level of behavioral disturbance (Aberrant Behavior Checklist, Community Edition; ABC-C) of this study’s subsample were similar to the full trial cohorts.

#### Passive viewing of emotional faces paradigm

A Tobii T120 infrared binocular eye tracker (Tobii Technology, Sweden) was used to collect gaze pattern data. This is an infrared video-based tracking system that monitors binocular eye movements employing a cornea reflection technique with a sampling rate of 120 Hz. The tracker is embedded in the computer monitor and is considered less invasive than head mounted units promoting more natural user behavior.

Stimuli were the same as those used in Farzin et al. [37, 38] and consisted of 60 color photographs of adult human faces from the NimStim Face Stimulus Set [45] and 60 scrambled versions of each facial image. Faces demonstrated a calm, happy, or fearful expression, with 20 trials of each. Faces and their corresponding scrambled image were displayed on a standard 50% gray background and matched on mean luminance. Stimuli were designed to imitate the size of an actual human face when viewed from a distance of 60 cm from the monitor (subtended a 12.12° by 17.19° region).

### Clinical assessments

As per the Novartis trial protocols, caregivers of all participants completed the ABC-C [46] throughout the trials to evaluate severity of aberrant behavior, and participants completed either the Stanford Binet Scale of Intelligence, Fifth Edition (for the adult trial; [47]) or the Leiter International Performance Scale, Third Edition (for the adolescent trial; [48]) at study entry, to examine the baseline cognitive profile of each cohort.

### Procedure

**Fig 1:**
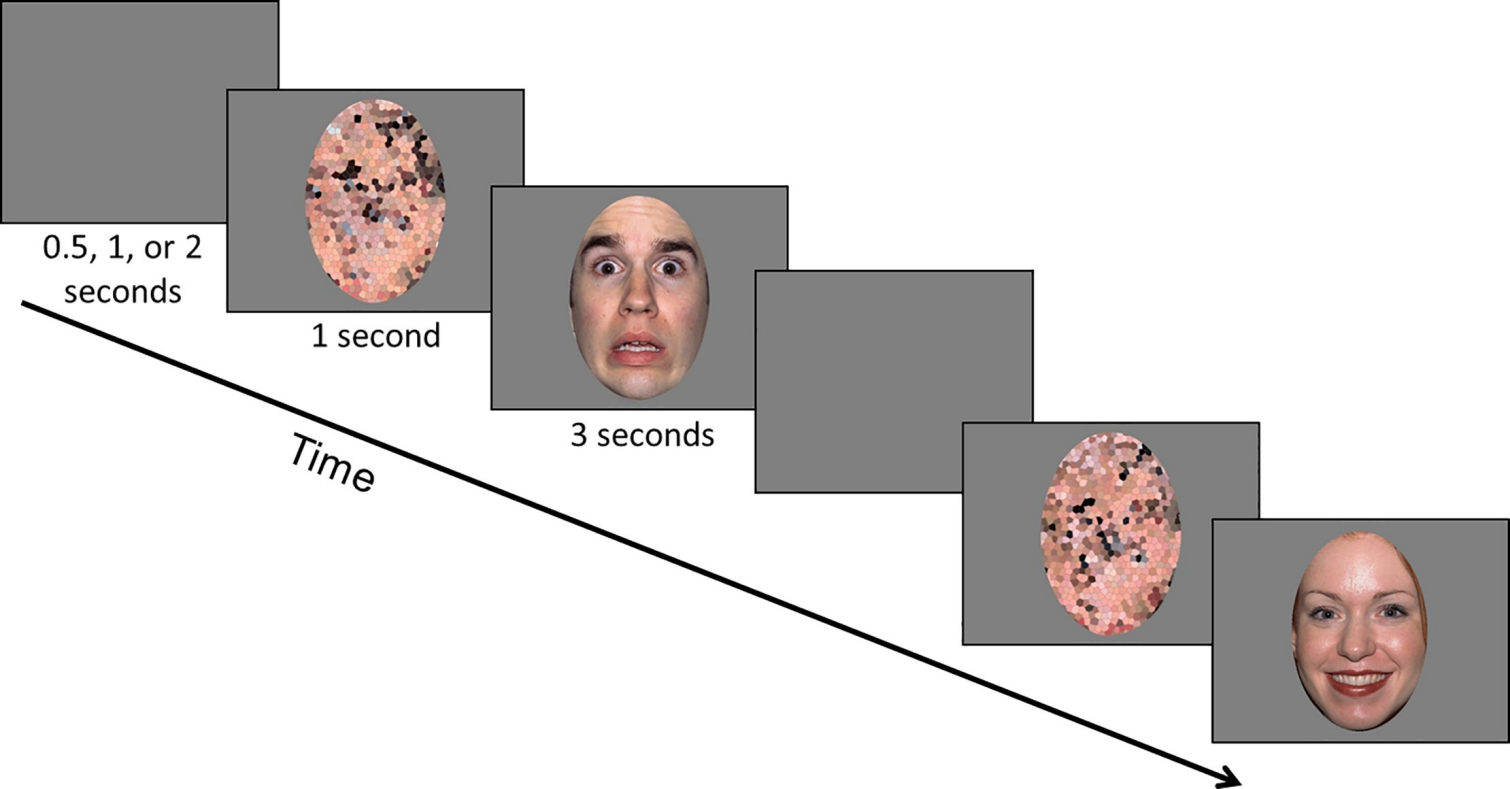
Eye tracking protocol. Participants viewed calm, fearful, or happy faces (random order) for 3 seconds each while eye gaze behavior and pupil size was recorded using a Tobii 120 Hz eye tracker. Each face was preceded by its scrambled version, matched on luminance and color pattern (as a control for pupil light reflex) examining pupil change associated with the social content and sympathetic nervous system activation. The primary area of interest was the eye region (including eyebrows) for examination of proportion of looking time and number of visual fixations to that region. The individuals in this figure have given written informed consent to publish the image (https://macbrain.org/resources.htm).

The eye tracking data was collected during passive viewing of the stimuli in a quiet room with the lights turned off. At the beginning of each eye tracking session a standardized 9-point calibration was completed. With an experimenter in the room and out of view, participants were asked to sit quietly, remain as still as possible, and watch the pictures presented on the screen. Given sensory reactivity and movement restriction challenges inherent to FXS, it was not possible to precisely standardize the distance from eyes to the screen by fixing head position; however position was continuously monitored using the track status feature of Tobii Studio and reminders to the participants to re-position were used as needed. Trials consisted of the presentation of a scrambled face for 1 second, followed by its matching face for 3 seconds (Fig 1). The order of these trials was pseudorandomized. Between each of the 60 trials a uniform grey screen was shown and the duration was randomly determined based on one of three time intervals; 0.5, 1, or 2 seconds.

### Data analysis

We were specifically interested in the total absolute looking time and fixations to the eye region. Total amount of time gazing anywhere on the face was a sum across regions (eyes, mouth, nose, other) of the average across trials in which the participant gazed at the specific region of the face. A fixation was defined as any data point within a 30 pixel radius that was recorded for at least 150 milliseconds, using the Tobii Fixation Filter. Total absolute looking time to the eye region and the number of fixations to the eye region were both summarized at the level of emotion for each time point and each participant, for a maximum of six observations per person. The mean number of observations was 5.8 (SD = 0.7). For the pupil diameter outcome, each emotion was summarized across trials for 11 intervals at each time point, for a maximum of 66 observations per participant. The mean number of pupil diameter measurements was 62 (SD = 11).

As described in Farzin et al. [37], pupilometry data was quantified as the mean pupil diameter for 250 millisecond intervals across the 3-seconds the stimuli was presented (excluding the last interval during which the screen transitioned to the next stimuli) for a total of 11 intervals. Pupilometry data was first filtered to remove values associated with blinks, loss of tracking the pupils by the eye tracker, or large changes in head position and then was averaged across both eyes. To compute pupil response to face-specific stimuli “standardized” by the average pupil size during the corresponding scrambled face the following calculation was used:

$$\frac{Pupil\;Size\;during\;Emotional\;Face–Pupil\;Size\;during\;Scrambled\;Face}{Pupil\;Size\;during\;Scrambled\;Face}$$

Analyses were conducted using mixed effects regression models [49, 50] to account for the repeated measures design for each of the outcomes (total absolute looking to the eye region, number of fixations to the eyes, and pupil response); all measures for each outcome across emotions and across both baseline and follow-up (repeated measures) were used as the dependent variables in the model. A square root transformation was applied to all fixation data due to positive skew to better meet the assumption of constant variance. For the outcomes total absolute looking time and fixation to the eyes, treatment group (placebo, 25mg mavoglurant, 50 mg mavoglurant, or 100 mg mavoglurant), emotional valence of stimuli (calm, happy, fearful), time, and corresponding interactions were considered in the primary analyses. Models further included random effects for study participant nested within site and time. Akaike information criterion was used for model building. After we determined the best fitting model from those tested, restricted maximum likelihood (REML) was used to estimate model parameters.

The trajectory of relative change in pupil size over the 3-second stimulus presentation was modeled using a non-linear growth curve model. Based on the shape of pupilometry trajectories observed in Farzin et al. [37], we began by testing unconditional linear and quadratic growth curve models to estimate the overall shape of pupil changes during stimulus presentation across all individuals, as well as all emotional valence and treatment conditions. This second unconditional model could account for quadratic change. In the linear model, time was expressed as the interval since the onset of stimulus presentation (interval). Interval since stimulus presentation squared (interval^2^) was added to the unconditional linear model to test for the curvilinear shape of this trajectory. After determining the form of the best fitting unconditional growth curve model we tested the influence of treatment, stimulus emotion, and their interaction in the primary analyses. Random effects in these models included study participant nested within site and interval. Akaike information criterion was used for model building.

To aid in the interpretation of effects, eye gaze outcomes at both time points were standardized by subtracting the mean and dividing by the standard deviation of the outcome at baseline in the calm condition across all treatment groups; for the pupilometry outcome, the data from the first interval was used. Coefficients in the models are then interpreted in terms of standard deviation units. Although on average participants were attentive to stimuli (looking at each trial for an average of about 2.75 seconds of the 3 seconds of possible looking time), some participants were much less attentive. We identified participants who provided gaze data for fewer than 7 trials per emotion for exclusion in sensitivity analyses. Secondary analyses for all outcomes considered the effects of including body mass index, race/ethnicity (Caucasian vs. non-Caucasian), and baseline ABC-C raw scores in the models. Results from the sensitivity and secondary analyses were similar to the primary analyses, so only results from the primary analyses are presented. Follow-up analyses investigated the effect of concomitant psychoactive medication use on results.

All analyses were conducted in SAS version 9.4 and a p-value less than 0.05 was considered statistically significant. To account for multiple comparisons between groups across the outcomes, we applied the Benjamini-Hochberg False Discovery Rate to all reported p-values and found that they all remained significant; we therefore only report the uncorrected p-values.

## Results

### Participant demographics

Participant baseline descriptive data are shown in Table 1. There were no significant differences in age, gender, race, *FMR1* methylation status, body mass, IQ, overall behavioral problems or total looking time to stimuli at baseline between those randomized to placebo or any of the three mavoglurant doses. None of the participants were taking anti-convulsants. Two individuals (both in the 50mg group) were taking alpha agonists. Anti-psychotics (n = 7, 2 in the 50mg group, 1 in the 100mg group and 4 in the placebo group), selective serotonin reuptake inhibitors (SSRI; n = 11, 1 in the 25mg group, 4 in the 50mg group, 3 in the 100mg group, and 3 in the placebo group), and stimulants (n = 10, 2 in the 25mg group, 3 in the 50mg group, 4 in the 100mg group, and 1 in the placebo group) were slightly more common, although the percentage of individuals on these medications did not differ between the groups.

**Table 1:** Participant descriptive information by treatment group.

	Placebo	25 mg	50mg	100 mg	*F*(*df*, *p*) or *p*
*N*	18	11	12	16	
Age (*M*, *SD*)	19.58 (6.60)	23.60 (7.19)	21.81 (7.87)	19.09 (5.16)	1.30 ((3,53),.28)
Gender (% male)	94.4	90.9	91.7	87.5	.92
Race (% non-Caucasian)	11.1	27.3	16.7	6.2	.52
Methylation (% fully methylated)	38.9	36.4	41.7	31.2	.96
Baseline BMI in kg/m^2^ (*M*, *SD*)	23.87 (7.32)	27.43 (4.65)	26.15 (3.87)	23.69 (4.28)	1.48 ((3, 53), .23)
Baseline IQ Score (*M*, *SD*)	41.39 (6.26)	40.00 (6.05)	44.42 (7.22)	44.69 (9.97)	1.2 ((3,53),.32)
Baseline ABC-C_FX_ Total Score (*M*, *SD*)	59.28 (26.27)	49.45 (24.60)	45.25 (28.58)	42.25 (22.96)	1.42 ((3,53),.25)
Baseline Total Looking Time (Min.; *M*, *SD*)	2.79 (1.45)	2.44 (1.12)	2.70 (1.04)	2.99 (1.48)	0.37 ((3,52),.78)

### Eye tracking data acquisition by group

All eye gaze data were summarized across up to 60 trials. At baseline, there was no difference in the proportion of trials in which eye gaze data were captured across the groups [*p* = .9; placebo: 80% of trials (SD = 25%); 25mg: 85% (12%); 50mg: 89% (12%); 100mg: 83% (20%)]. Similarly, there was no difference in the proportion of trials with eye gaze data across groups [*p* = .8; placebo: 84% (14%); 25mg: 82% (17%); 50mg: 86% (10%); 100mg: 86% (17%)].

### Absolute looking time to the eye region

**Fig 2:**
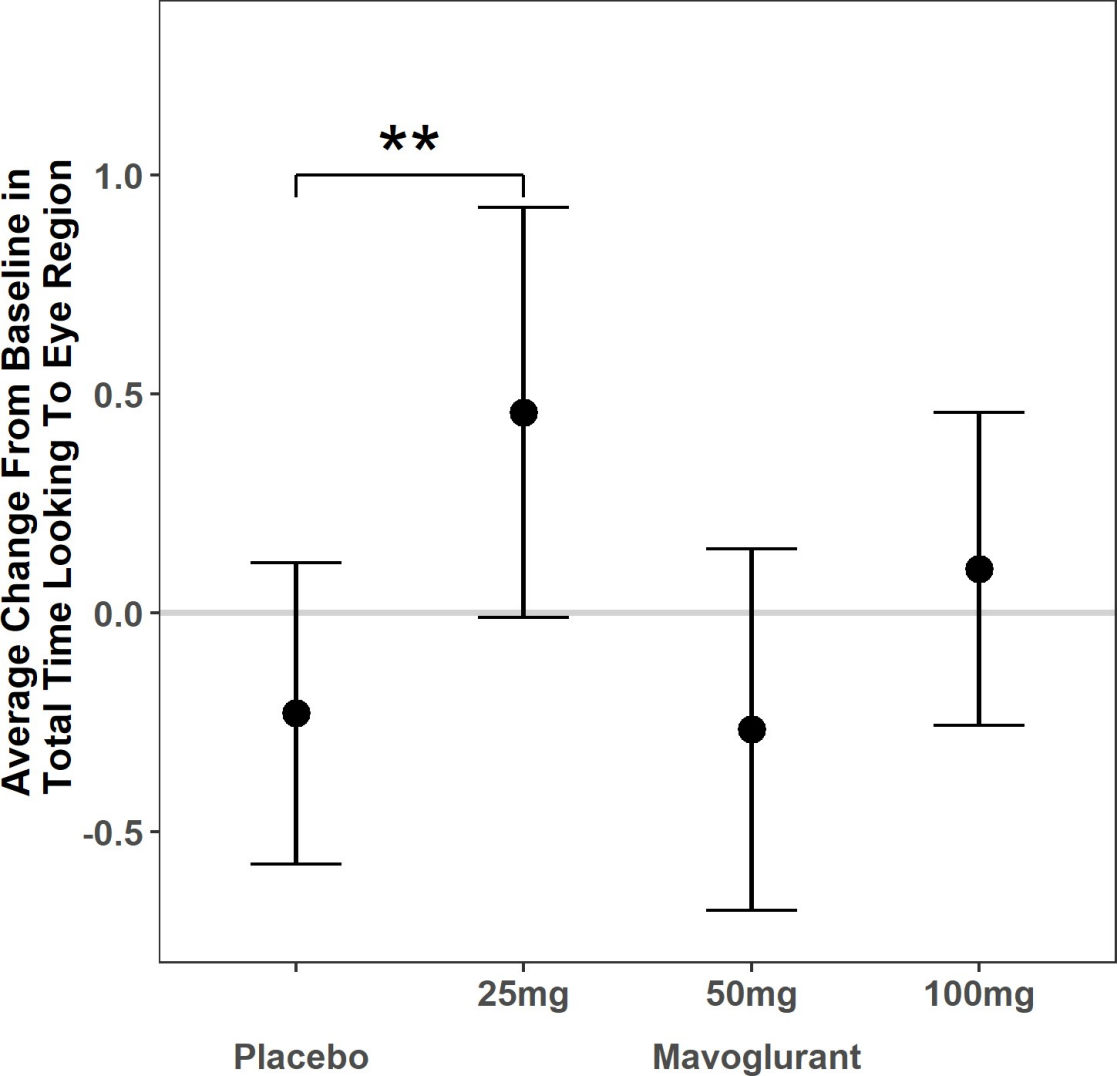
Absolute looking time to the eye region. Average change from baseline in total absolute looking time to the eye region of faces by adolescent and adult patients with fragile X syndrome following 3 months of treatment with placebo vs. 25 mg, 50 mg, or 100 mg of the mGluR5 negative modulator mavoglurant. Dots reflect the model estimated change from baseline for each group in standard deviation units. Bars reflect 95% confidence intervals. Horizontal line at zero reflects no estimated change. **Those treated with 25 mg of mavoglurant experienced greater change from baseline on average than the placebo group (p<0.01).

When comparing between the groups, there was no change in total looking time to faces overall in the placebo group [β = -0.28, standard error (SE) = 0.37, *p* = .2] and no significant mean difference [F(3,50) = 1.56, *p* = .2] in change in total looking time to faces overall between the groups. The linear mixed model with total absolute looking time to the eye region specifically comparing change in the mavoglurant dosage groups to placebo (see Table 2) yielded a significant effect of treatment, such that those treated with 25mg mavoglurant showed a 0.69 standard deviation (SD) increase in looking to the eye region at follow-up compared to baseline relative to the placebo group [β = 0.69, SE = 0.29, *p* = .02, 95% confidence interval (CI) = (0.11, 1.27)]. There was no significant difference in amount of change for individuals in the 50 mg or 100 mg groups relative to the placebo group (Fig 2, S1 Table).

**Table 2:** Associations of mavoglurant treatment with change in absolute looking time to the eye region.

Random Effects	Variance				
Participant:Site	0.84				
Time	0.45				
Residual	0.12				
Fixed Effects	Estimate	SE	df	t value	p value
Time	-0.23	0.17	50	-1.31	.19
25 mg dose*time	**0.69**	**0.29**	**220**	**2.33**	**.02**
50 mg dose*time	-0.04	0.27	220	-0.14	.89
100 mg dose*time	0.33	0.25	220	1.31	.19

Model includes effects of emotion and treatment group on baseline level.

### Fixations to the eye region

**Fig 3:**
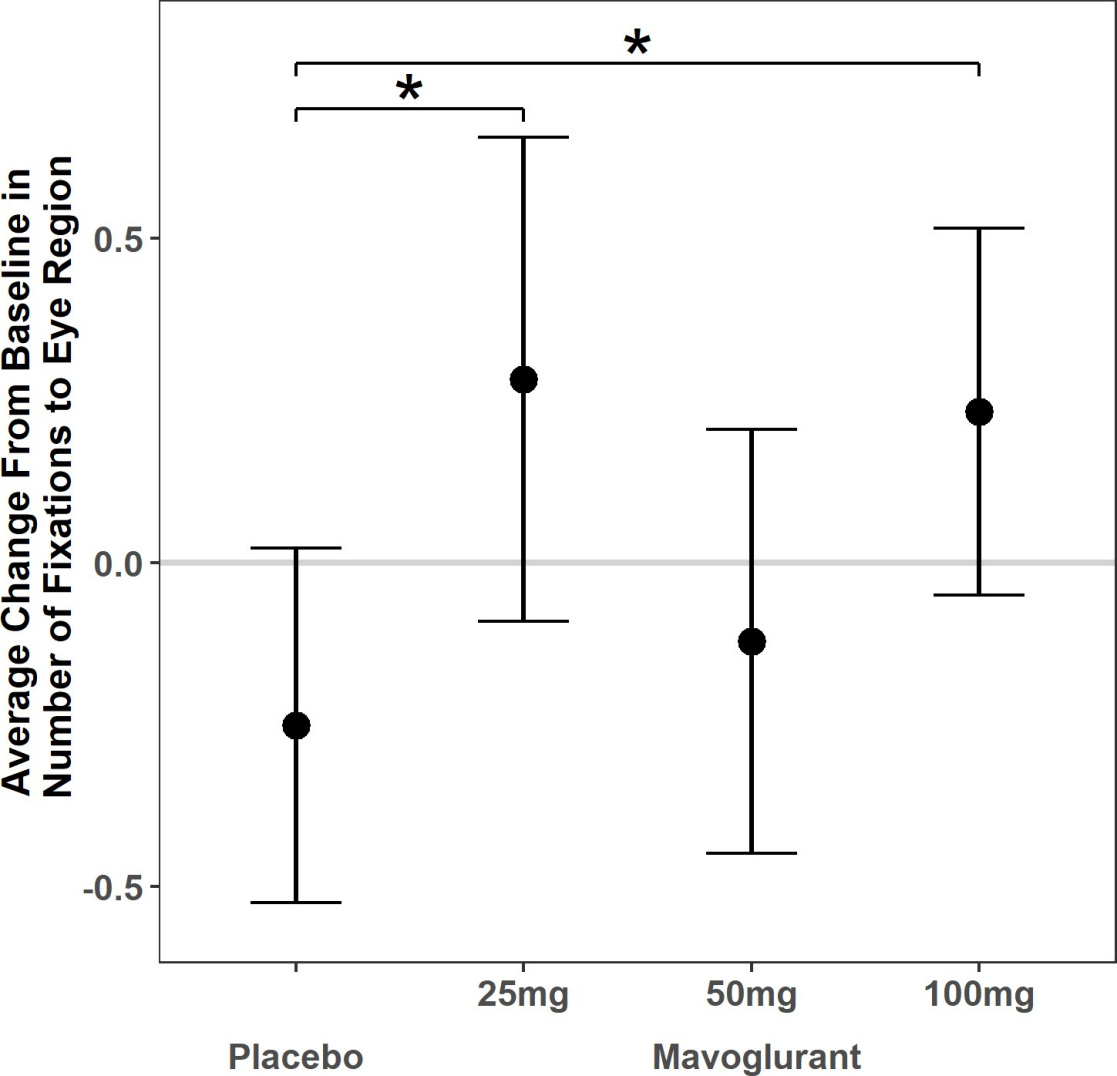
Number of fixations. Average change in number of fixations to the eye region of faces by adolescent and adult patients with fragile X syndrome following 3 months of treatment with placebo vs. 25 mg, 50 mg, or 100 mg of the mGluR5 negative modulator mavoglurant. Dots reflect the model estimated change for each group in standard deviation units. Bars reflect 95% confidence intervals. Horizontal line at zero reflects no estimated change. *Those treated with 25 mg or 100mg of mavoglurant experienced more change on average than the placebo group (p<0.05).

The linear mixed model with fixations to the eye region comparing mavoglurant dosage groups to placebo (see Table 3) yielded a significant difference in the amount of change, such that the 25 mg and 100 mg groups increased about 0.5 SD more than the placebo group (25 mg: β = 0.53, SE = 0.23, *p* = .02, 95% CI = (0.07, 1.00); 100 mg: β = 0.48, SE = 0.20, *p* = .02, 95% CI: (0.09, 0.88)). There was no significant difference in amount of change between the 50 mg group and the placebo group (Fig 3, S2 Table).

**Table 3:** Associations of mavoglurant treatment with change in number of fixations to the eye region.

Random Effects	Variance				
Participant:Site	0.84				
Time	0.22				
Residual	0.17				
Fixed Effects	Estimate	SE	df	t value	p value
Time	-0.25	0.14	50	-1.81	.08
25 mg dose*time	**0.53**	**0.23**	**220**	**2.27**	**.02**
50 mg dose*time	0.13	0.21	220	0.60	.55
100 mg dose*time	**0.48**	**0.20**	**220**	**2.42**	**.02**

Model includes effects of emotion and treatment group on baseline level.

### Pupil reactivity to faces

**Fig 4:**
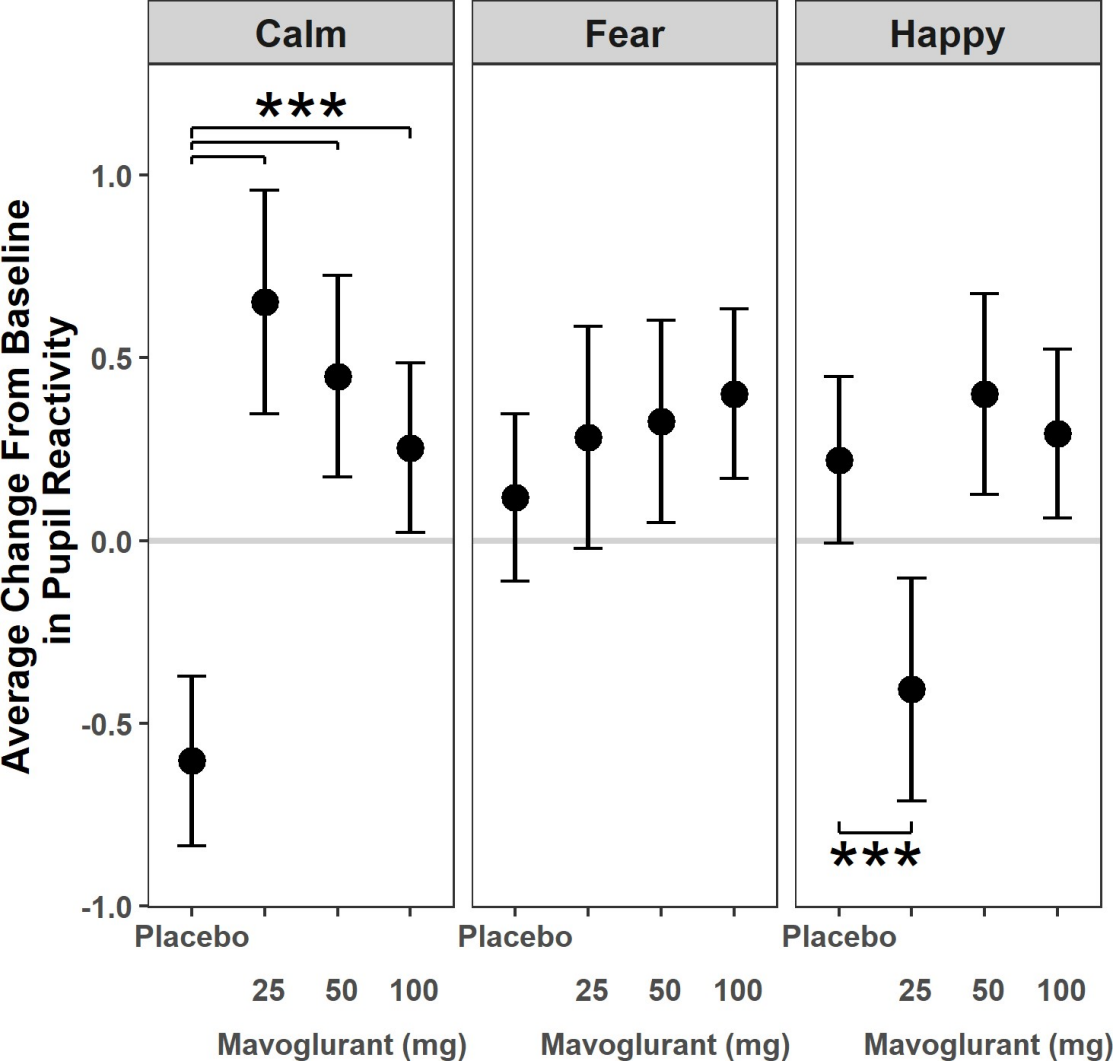
Pupilometry. Sympathetic nervous system-mediated pupil reactivity to calm and emotional faces in patients with fragile X syndrome treated for 3 months with placebo or 25 mg, 50 mg, or 100mg mGluR5 negative modulator mavoglurant. Dots reflect the model estimated change for each group in standard deviation units. Bars reflect 95% confidence intervals. Horizontal line at zero reflects no estimated change. ***Those treated with 25 mg, 50 mg or 100mg of mavoglurant experienced more change on average than the placebo group in the Calm condition (p<0.001). In addition, those treated with 25 mg of mavoglurant had less reactivity than the placebo group in the Happy condition (p<0.001).

The quadratic growth curve models of pupil change during exposure to emotional faces demonstrated an overall downward concave shape at both baseline and follow-up (interval: β = 0.17, SE = 0.03, t = 5.72, *p*<0.001; interval^2^: β = -0.007, SE = 0.002, t = -3.17, *p* = 0.002) with significant differences in amount of change by emotional stimulus and treatment group; there were no differences in overall shape between baseline and follow-up (see Table 4). In contrast to models examining fixations and looking time, emotion had a significant effect on pupil reactivity over time, such that, relative to calm, both fearful (β = 0.72, SE = 0.17, t = 4.35, *p* <0.001) and happy faces (β = 0.82, SE = 0.16, t = 4.98, *p* < 0.001) elicited 0.7–0.8 SD more pupil dilation in the placebo condition, compared to baseline. Mavoglurant treatment resulted in 0.9–1.3 SD greater pupil dilation at follow-up in the calm condition relative to the placebo group (25mg: β = 1.26, SE = 0.20, t = 6.41, *p*<0.001, 95% CI = (0.87, 1.64); 50mg: β = 1.05, SE = 0.18, t = 5.73, *p*<0.001, 95% CI = (0.69, 1.41); 100mg: β = 0.86, SE = 0.17, t = 5.12, *p*<0.001, 95% CI = (0.53, 1.19)). However, 25mg mavoglurant treatment resulted in significantly less change in pupil reactivity than the placebo group in the happy condition (β = -0.63, SE = 0.19, t = -3.23, *p* = 0.001, 95% CI = (-1.01, -0.25)). See Fig 4 and S3 Table.

**Table 4:** Associations of mavoglurant treatment and exposure to emotional faces with pupil diameter change.

Fixed Effects	Estimate	SE	df	t	p value
Time	-0.60	0.12	3376	-5.09	< .001
25 mg Mavoglurant*time	**1.26**	**0.20**	**3376**	**6.41**	**< .001**
50 mg Mavoglurant*time	**1.05**	**0.18**	**3376**	**5.73**	**< .001**
100 mg Mavoglurant*time	**0.86**	**0.17**	**3376**	**5.12**	**< .001**
Fear*time	**0.72**	**0.17**	**3376**	**4.35**	**< .001**
Happy*time	**0.82**	**0.16**	**3376**	**4.98**	**< .001**
25 mg Mavoglurant* Fear*time	**-1.09**	**0.27**	**3376**	**-3.98**	**< .001**
50 mg Mavoglurant* Fear*time	**-0.84**	**0.26**	**3376**	**-3.26**	**< .001**
100 mg Mavoglurant* Fear*time	**-0.57**	**0.24**	**3376**	**-2.43**	**.01**
25 mg Mavoglurant* Happy*time	**-1.88**	**0.27**	**3376**	**-6.86**	**< .001**
50 mg Mavoglurant* Happy*time	**-0.87**	**0.26**	**3376**	**-3.38**	**< .001**
100 mg Mavoglurant* Happy*time	**-0.78**	**0.23**	**3376**	**-3.34**	**< .001**

Model includes effects of interval, interval^2^, treatment group, emotion, and the treatment group by emotion interaction on baseline level. There was no interaction between interval or interval^2^ and time.

### Effect of concomitant psychoactive medications

Based on the inconsistent, or non-linear results across dosages of mavoglurant, the influence of concomitant psychoactive medications (CPM) was assessed as a possible explanation. Due to small sample sizes in different medication classes, medication use was collapsed across CPM. Differences in rate of change between dosages of mavoglurant and placebo varied by CPM for total absolute looking time to the eye region (p = .001) and number of fixations to the eye region (p = .001), but not pupil reactivity to faces (p = .67). For total absolute looking time to the eye region, within the 25mg group the sample size was too small in the CPM subgroup (n = 3) to allow any valid follow-up statistical comparisons, however visual review of individual patterns of change suggested that these individuals (1 with SSRI, 2 with stimulant) showed more substantial increases in looking to the eye region compared to those not taking CPM. No significant effects of CPM were found in the other two dosage groups. When considering the fixations to the eye region, within the 25 mg group, the same 3 participants taking CPM appeared to improve more than those not taking CPM. In contrast, those in the 100mg group not taking CPM (n = 8) improved more than those taking CPM [β = -0.59, SE = 0.26, *p* = .02, 95% CI = (-1.10, -0.09)] and those not taking CPM improved more than those in the placebo group not taking CPM [β = 0.92, SE = 0.24, *p* < .001, 95% CI = (0.44, 1.40)]. Also, those taking CPM in the 100 mg group did not improve more than the placebo group taking CPM [β = -0.01, SE = 0.26, *p* = .96, 95% CI = (-0.53, 0.51)].

## Discussion

We used a laboratory-based eye tracking paradigm to demonstrate that mavoglurant, a negative allostatic modulator of mGluR5 activity, significantly improves visual attention to the eyes in adults and adolescents with FXS relative to placebo in the context of a controlled trial. The improvement in eye gaze as a result of mavoglurant treatment in FXS may be driven by decreases in levels of social anxiety. In fact, the seminal paper introducing the mGluR theory of fragile X [27] highlighted that MPEP (a selective mGlur5 negative modulator) has broad anticonvulsant and anxiolytic effects. Two studies documented that negative modulation of mGluR5 activity normalizes social behavior in the *Fmr1* knockout (KO) mouse [51, 52]. Gantois et al [52] used a three-chambered task to determine sociability and preference for social novelty and showed that mavoglurant was able to restore sociability behavior of KO mice to levels of wild type littermates. De Esch et al [51] used the Automated Tube Test to demonstrate partial rescue of altered social behavior of KO mice, using both genetic (mGluR5 deletion heterozygotes) and pharmacological (mavoglurant) inhibition. Later, Suvrathan and colleagues [53] demonstrated that amygdala long term potentiation (LTP) is impaired in these mice and rescued by MPEP. The amygdala LTP abnormalities observed in *Fmr1* KO mice may be consistent with human brain functional MRI (fMRI) data demonstrating increased sensitization of the amygdala with repeated exposure to direct human gaze in patients with the disorder [40]. Another fMRI investigation of face processing in FXS showed a very strong association between fusiform gyrus hypoactivation and gaze to the eye region, but enhanced activation in hippocampus, insula, and superior temporal sulcus [43]. Thus the phenotypic response to faces in FXS appears to be abnormally regulated by a diverse network of activity in regions known to modulate social behavior and emotional responses. Together, these data suggest that mavoglurant could at least partially normalize this network’s response to social stimuli in FXS, leading to detectible changes in eye gaze behavior. Certainly, direct measurement of brain activity changes tied to social stimuli, related to targeted treatment in FXS would help to substantiate our findings. Eye tracking will be further evaluated as a key biomarker in a study to be conducted through the NeuroNext network, assessing the effects of mavoglurant on language learning in young children with FXS (clinicaltrials.gov, NCT 02920892). Similar findings in the NeuroNext trial would replicate and help substantiate the findings in this study.

Although the sample sizes by mavoglurant dose are not large, it is interesting to note that the lower dose group (for absolute looking time to the eye region as well as pupil reactivity) showed as much or more change as the higher dose groups. Our follow-up analyses examining the potential effect of concomitant medication use suggested that the 3 participants on 25 mg mavoglurant and additional psychoactive medications showed more improvement in looking duration and fixations within the eye region than those not taking these additional medications. Given the size of this subgroup, the observations might be idiosyncratic to these individuals and attributed to chance. In the 100 mg (highest) dose group, however, the improvement on fixations to the eye region compared to placebo was significantly more robust in those not taking concomitant medications. This might indicate that the higher dose of mavoglurant has a larger impact on eye gaze behavior in FXS, and that detection of effects of treatment may be more difficult or confounded by mixed influence of different concomitant medications. This latter interpretation, if confirmed by other studies, could have implications for trial design, and might warrant re-analysis of the effects of concomitant medications on the clinical outcomes in the larger multi-center trial of mavoglurant. Indeed, the potential impact and distributions of concomitant medications across trial groups were not reported in the previously published study [2]. However we caution that the sample sizes in the present study are probably not adequate to conclude with confidence the relative improvements associated with different doses of mavoglurant, and the variation in effects by dose may in fact be due to chance. Despite the nuances of dose seen here, the overall results of the study appear to support the conclusion that mavoglurant (at doses proven to occupy the targeted receptors in humans) had some effect on eye gaze behavior and pupil reactivity to emotional stimuli in these patients.

In the context of these mavoglurant trials, the eye tracking measures appear to be more sensitive to treatment than the clinical measures of aberrant behavior and social responsiveness reported by caregivers (as these showed no significant improvement over placebo in the larger cohorts [2]. As such they may provide an approach to determining whether a targeted treatment aimed at reversing specific neural deficits has engaged its target and resulted in syndrome-relevant and CNS-mediated changes. The increased sensitivity of the eye tracking measures may be due to the relative objectivity of measurement (compared to rating scales), less prominent placebo effects, high-resolution and repeated measurements contributing to increased reliability and power, and the narrow focus on a well-described phenotypic feature of the disorder.

The pupilometry results revealed relatively robust (in terms of effect sizes) and highly significant changes related to mavoglurant treatment. Given that our prior studies showed heightened pupil reactivity to emotional faces in FXS, we expected mavoglurant would dampen this reaction, as might be predicted from the *Fmr1* KO mice and human fMRI studies summarized above. In fact, this treatment appears to contribute to greater overall pupil dilation during exposure to these social stimuli, mainly driven by reactivity to calm faces. We were unable to find any clear correlation between pupil reactivity and clinical changes; thus it is difficult to interpret this finding. We speculate that individuals may become initially more sensitized to the social and emotional qualities of the stimuli as a result of treatment, leading to stronger sympathetic responses measured by pupil change. The effect with calm faces in particular is interesting, and might be related to enhanced processing of or reactivity to these more subtle and ambiguous social stimuli.

### Limitations

While the eye tracking protocol used in this study affords experimental control and precision of measurement, this passive task is limited to response to stimuli on a computer screen and may not accurately reflect social gaze deficits in the individual’s normal environment. Also, we emphasize that the eye gaze measures do not represent clinical outcomes and are probably not appropriate to use as primary endpoints in controlled trials. Rather, they might be considered biobehavioral markers that can be useful for indexing target engagement and early treatment response. Finally, the participants in the current study were only a subset of the larger trial, and when divided by dose groups, the power to detect differences is reduced and probability of type I and II errors is increased, and generalization to all the participants in the larger trials is less clear.

## Conclusions

In summary, we show that eye gaze behavior and sympathetic nervous system responsiveness to social-emotional stimuli are altered by mGluR5 modulation in patients with FXS, providing evidence that sensitive laboratory-based biobehavioral measures can be useful tools for detecting targeted treatment-related responses that may not be identified by broader clinical assessments over short time frames of several months. It is our hope that these findings will help to guide future clinical trials by showing the potential for mGluR5 negative modulators to modify human behavior, and emphasizing the importance of syndrome-specific and physiological outcome measurement development for assessing target engagement, defining participant selection criteria and helping determine what agents should best be explored in larger and longer trials with clinical outcomes for neurodevelopmental disorders.

## Supporting information

S1 Table Supplemental Table 1. Total absolute looking time to the eye region by group, emotion, and time point.
(DOCX)

S2 Table Supplemental Table 2. Fixations to the eye region by group, emotion, and time point.
(DOCX)

S3 Table Supplemental Table 3. Pupil reactivity, averaged across intervals, by group, emotion, and time point.
(DOCX)

S1 Data Absolute looking time data. (CSV)

S2 Data Fixation number data. (CSV)

S3 Data Pupilometry data. (CSV)
