# Supplementary material for: Effects of mavoglurant on visual attention and pupil reactivity while viewing photographs of faces in Fragile X Syndrome
Source: PLoS One. 2019 Jan 17;14(1):e0209984. doi: 10.1371/journal.pone.0209984 (PMC6336311; doi:10.1371/journal.pone.0209984)
Supplement: S1 Table — Total absolute looking time to the eye region by group, emotion, and time point. (DOCX) [file pone.0209984.s001.docx]

**Supplemental Table 1. Total absolute looking time to the eye region by group, emotion, and time point**

|  | Baseline | | | | Follow-up | | | |
| --- | --- | --- | --- | --- | --- | --- | --- | --- |
| Emotion | Placebo  (n=18) | 25mg  (n=10) | 50mg  (n=12) | 100mg  (n=16) | Placebo  (n=17) | 25mg  (n=10) | 50mg  (n=12) | 100mg  (n=16) |
| Calm | 705.6 (435.9) | 392.9 (240.3) | 408.7 (317.3) | 748.7 (613.0) | 607.9 (367.0) | 612.2 (685.7) | 331.5 (185.7) | 816.6 (468.8) |
| Happy | 643.8 (457.8) | 394.6 (299.1) | 457.7 (375.9) | 687.3 (531.5) | 552.4 (373.1) | 506.2 (510.5) | 272.4 (174.5) | 774.7 (534.0) |
| Fear | 721.5 (480.8) | 448.2 (284.8) | 466.4 (366.8) | 789.4 (644.3) | 614.4 (319.8) | 690.9 (684.1) | 356.7 (241.8) | 775.1 (459.4) |

At baseline, there were no significant differences by group for any emotion (calm: p=0.08, happy: p=0.3, fear: p=0.2)
