# Supplementary material for: Effects of mavoglurant on visual attention and pupil reactivity while viewing photographs of faces in Fragile X Syndrome
Source: PLoS One. 2019 Jan 17;14(1):e0209984. doi: 10.1371/journal.pone.0209984 (PMC6336311; doi:10.1371/journal.pone.0209984)
Supplement: S2 Table — Fixations to the eye region by group, emotion, and time point. (DOCX) [file pone.0209984.s002.docx]

**Supplemental Table 2. Fixations to the eye region by group, emotion, and time point**

|  | Baseline | | | | Follow-up | | | |
| --- | --- | --- | --- | --- | --- | --- | --- | --- |
| Emotion | Placebo  (n=18) | 25mg  (n=10) | 50mg  (n=12) | 100mg  (n=16) | Placebo  (n=17) | 25mg  (n=10) | 50mg  (n=12) | 100mg  (n=16) |
| Calm | 1.8 (1.0) | 1.2 (0.6) | 1.1 (0.8) | 1.8 (1.3) | 1.6 (1.0) | 1.5 (1.0) | 1.0 (0.6) | 2.1 (1.2) |
| Happy | 1.8 (1.1) | 1.2 (0.7) | 1.1 (0.9) | 1.7 (1.0) | 1.5 (1.1) | 1.3 (0.7) | 0.9 (0.6) | 1.9 (1.2) |
| Fear | 2.0 (1.1) | 1.4 (0.7) | 1.1 (0.7) | 1.8 (1.2) | 1.7 (1.0) | 1.7 (1.1) | 1.0 (0.6) | 2.1 (1.0) |

At baseline, there were no significant differences by group for any emotion (calm: p=0.1, happy: p=0.2, fear: p=0.1). We note that these summary statistics are presented for the actual number of fixations, while the analyses presented in the paper utilize the square root of the number of fixations to better meet the assumptions of our analytic methods.
