# Supplementary material for: Effects of mavoglurant on visual attention and pupil reactivity while viewing photographs of faces in Fragile X Syndrome
Source: PLoS One. 2019 Jan 17;14(1):e0209984. doi: 10.1371/journal.pone.0209984 (PMC6336311; doi:10.1371/journal.pone.0209984)
Supplement: S3 Table — Pupil reactivity, averaged across intervals, by group, emotion, and time point. (DOCX) [file pone.0209984.s003.docx]

**Supplemental Table 3. Pupil reactivity, averaged across intervals, by group, emotion, and time point**

|  | Baseline | | | | Follow-up | | | |
| --- | --- | --- | --- | --- | --- | --- | --- | --- |
| Emotion | Placebo  (n=17) | 25mg  (n=10) | 50mg  (n=11) | 100mg  (n=16) | Placebo  (n=16) | 25mg  (n=9) | 50mg  (n=12) | 100mg  (n=16) |
| Calm | 0.008 (0.03) | -0.003 (0.03) | -0.002 (0.02) | 0.007 (0.02) | -0.006 (0.04) | 0.01 (0.03) | 0.01 (0.03) | 0.01 (0.02) |
| Happy | 0.009 (0.03) | 0.01 (0.01) | 0.01 (0.03) | 0.001 (0.03) | 0.01 (0.03) | -0.0006 (0.02) | 0.02 (0.02) | 0.009 (0.02) |
| Fear | 0.001 (0.03) | -0.001 (0.02) | -0.01 (0.02) | -0.002 (0.02) | 0.006 (0.03) | 0.005 (0.02) | -0.003 (0.02) | 0.008 (0.02) |

At baseline, there were no significant differences by group for any emotion (calm: p=0.5, happy: p=0.7, fear: p=0.3)
